# The Comparative Sufficiency of ChatGPT, Google Bard, and Bing AI in Answering Diagnosis, Treatment, and Prognosis Questions About Common Dermatological Diagnoses

**DOI:** 10.2196/60827

**Published:** 2025-01-07

**Authors:** Courtney A Chau, Hao Feng, Gabriela Cobos, Joyce Park

**Affiliations:** 1 Icahn School of Medicine at Mount Sinai, New York, NY, United States; 2 Department of Dermatology, University of Connecticut Health Center, Farmington, CT, United States; 3 Department of Dermatology, Tufts Medical Center, 260 Tremont St, Fl 13, Boston, MA, 02116, United States, 1 617-636-0156; 4 Skin Refinery PLLC, Spokane, WA, United States

**Keywords:** artificial intelligence, AI, ChatGPT, atopic dermatitis, acne vulgaris, cyst, actinic keratosis, rosacea, diagnosis, treatment, prognosis, dermatological, patient, chatbot, dermatologist

## Abstract

Our team explored the utility of unpaid versions of 3 artificial intelligence chatbots in offering patient-facing responses to questions about 5 common dermatological diagnoses, and highlighted the strengths and limitations of different artificial intelligence chatbots, while demonstrating how chatbots presented the most potential in tandem with dermatologists’ diagnosis.

## Introduction

Artificial intelligence (AI) chatbots, such as ChatGPT, offer platforms for patients to ask medical questions, particularly with limited access to care [1]. Although ChatGPT utility in dermatology has been assessed, few studies have compared the performance between chatbots [2]. This study compared the clinical utility of the unpaid versions of ChatGPT 3.5, Google Bard, and Bing AI in generating patient-facing responses to questions about 5 common dermatological diagnoses (atopic dermatitis, acne vulgaris, actinic keratosis, cyst, and rosacea) [3].

## Methods

For each condition, 2 diagnosis, 2 treatment, and 1 prognosis questions were devised. Diagnosis questions requested a diagnosis and presented the patient history including age, sex, symptoms (duration/location), treatments and outcomes, and medical history. Nineteen questions were modeled from questions on Reddit forums (“r/AskDocs” and “r/dermatology”). For topics with insufficient Reddit questions, the coauthors devised prompts reflecting common questions in their experience (6 questions).

Questions were inputted into each chatbot; the prompts used are shown in Multimedia Appendix 1. Three board-certified dermatologists scored the responses on appropriateness for a patient-facing platform (Yes/No), sufficiency for clinical practice (Yes/No: not specific, not concise, or inaccurate information), accuracy from 1 (completely inaccurate) to 6 (completely accurate), and overall from 1 (worst possible answer) to 10 (best possible answer) [4]. The Wilcoxon rank-sum test was used for pairwise comparisons. *P*-values were adjusted using the Bonferroni correction.

## Results

**Figure 1.**
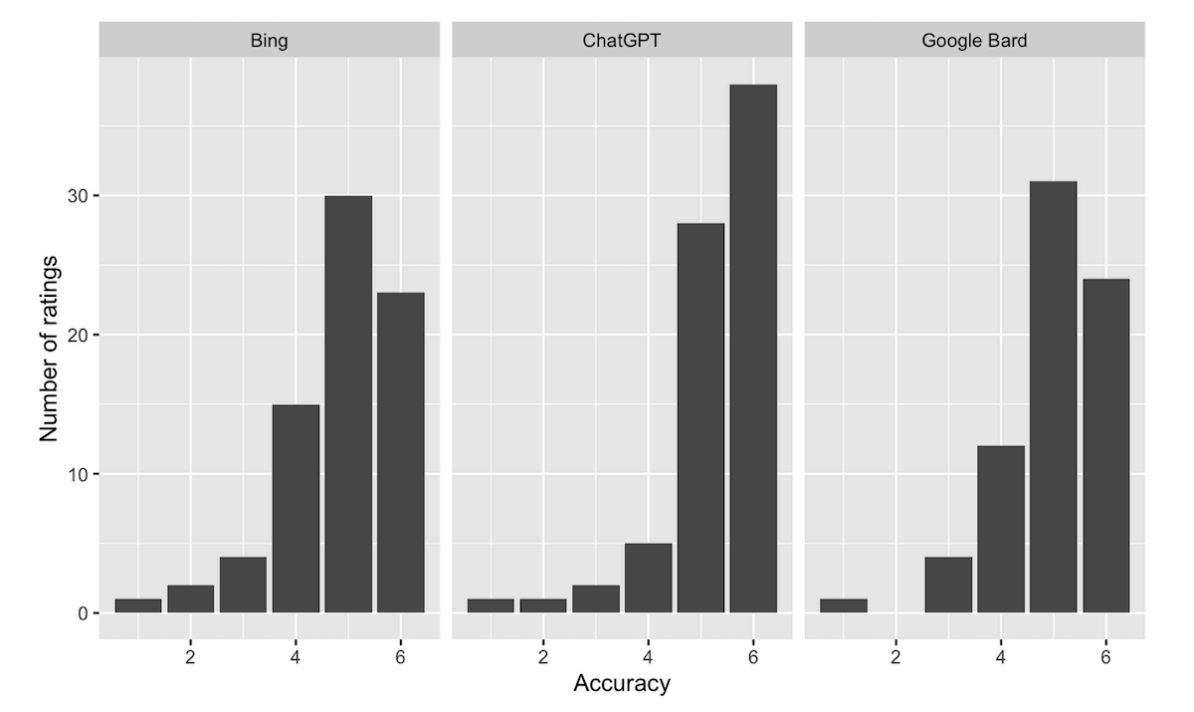
Distribution of the accuracy ratings for each chatbot. The accuracy scores from the three board-certified dermatologists ranged from 1 (completely inaccurate) to 6 (completely accurate).

One response was omitted because Google Bard declined answering the second atopic dermatitis diagnosis question (“I am a 19-year old…”), responding with, “I’m just a language model, so I can’t help you with that.” ChatGPT responses had significantly lower Flesch reading ease scores than Google Bard (*P*<.001) and Bing AI (*P*<.001), indicating lower comprehensibility (Table 1). ChatGPT responses received significantly higher accuracy (*P*=.01, Figure 1) and overall (*P*=.003) ratings than Bing AI. Considering patient-facing platform appropriateness and clinical practice sufficiency, ChatGPT received the most appropriate (95%) and sufficient (55%) ratings; Bing AI received the fewest (87% and 55%, respectively). In total, 45%, 49%, and 53% of ChatGPT, Google Bard, and Bing AI responses, respectively, had inaccurate information or were not specific. For diagnosis prompts, 9 of 10 of ChatGPT and Bing AI and 7 of 10 of Google Bard responses included the intended diagnosis. Of the 25 responses from each chatbot, 25 of Bing AI’s, 24 of ChatGPT’s, and 19 of Google Bard’s responses emphasized the importance of consulting healthcare professionals. No fabrication or hallucination was observed for any chatbot responses.

**Table 1.** Descriptive statistics of scores between chatbots.

	ChatGPT 3.5 (n=75)	Google Bard (n=72)	Bing AI (n=75)
Mean Flesch reading ease score (SD)*^a^	33.90 (8.1)	49.72 (15.4)	46.53 (9.7)
Mean accuracy (SD)	5.29 (0.97)	5.00 (0.98)	4.87 (1.1)
Mean overall rating (SD)	8.37 (1.8)	7.94 (1.9)	7.41 (2.1)
Number of responses appropriate for a patient-facing platform (%)	71 (95)	65 (90)	65 (87)
Sufficiency for clinical practice			
Yes (%)	41 (55)	35 (49)	35 (47)
No: not specific enough (%)	14 (19)	15 (21)	23 (31)
No: inaccurate information (%)	20 (27)	20 (28)	17 (23)
No: not concise (%)	0	2 (3)	0

a Out of n=25 for ChatGPT and Bing AI and n=24 for Google Bard because only 1 Flesch reading ease score was calculated for each response. The other measures in the table are based on evaluation of each chatbot response by 3 board-certified dermatologists.

## Discussion

ChatGPT outputs were most accurate and appropriate for patient questions. However, ChatGPT responses had college-level readability, limiting public utility [5]. Responses were deemed sufficient for clinical practice if the chatbot concisely provided completely correct information that specifically answered the patient’s question without missing critical components. Only approximately half the responses were sufficient for clinical practice, primarily due to inaccuracies and lack of specificity. ChatGPT and Bing AI performed the best at diagnosis and emphasized the importance of seeking input from a healthcare professional. Google Bard did not perform well in these domains, indicating that it is less suitable for suggesting diagnoses. Despite the better diagnostic performance of ChatGPT and Bing AI, an unranked list of conditions with differing treatments is not actionable for patients. Chatbots present more potential in offering advice once a diagnosis has been established. This study is limited by exploring only 5 questions for each of the 5 conditions. Exploring a broader range of conditions with a larger set of questions would more robustly capture chatbots’ performance. However, this study lays the groundwork for future research to compare chatbots using more expansive domains.

ChatGPT 3.5 displays more promise than Google Bard and Bing AI in evaluating, diagnosing, and suggesting a treatment plan for dermatologic conditions, consistent with previous findings, in which the chatbots’ responses to questions about melanoma were evaluated [2]. However, this study revealed several important improvements needed for all 3 chatbots: enhancing readability, removing inaccuracies, and improving information specificity. Dermatologists may be able to reference these AI in practice, to limited extents, by suggesting patients use AI as a reference only to obtain information about the condition after being diagnosed. This strategy is similar to paper handouts, where AI chatbots provide background knowledge that patients can later follow-up on with their dermatologist. In conclusion, while chatbot utility is most promising in tandem with a dermatologist’s diagnosis and contributes to information dissemination, chatbots should not function as a first-line independent entity. As access to AI grows, dermatologists must be aware of the quality of information patients may receive from AI and how it may differ from a dermatologist’s advice.

## Supplementary material

10.2196/60827 Multimedia Appendix 1 Prompts inputted into ChatGPT 3.5, Google Bard, and Bing AI.

## Abbreviations

AI: artificial intelligence

## References

[1] Baker MN, Burruss CP, Wilson CL (2023). ChatGPT: a supplemental tool for efficiency and improved communication in rural dermatology. Cureus.

[2] Mu X, Lim B, Seth I (2024). Comparison of large language models in management advice for melanoma: Google’s AI BARD, BingAI and ChatGPT. Skin Health Dis.

[3] Landis ET, Davis SA, Taheri A, Feldman SR (2014). Top dermatologic diagnoses by age. Dermatol Online J.

[4] Young JN, Poplausky D (2023). The utility of ChatGPT in generating patient-facing and clinical responses for melanoma. J Am Acad Dermatol.

[5] Hutchinson N, Baird GL, Garg M (2016). Examining the reading level of internet medical information for common internal medicine diagnoses. Am J Med.

