# Supplementary material for: The Comparative Sufficiency of ChatGPT, Google Bard, and Bing AI in Answering Diagnosis, Treatment, and Prognosis Questions About Common Dermatological Diagnoses
Source: JMIR Dermatol. 2025 Jan 7;8:e60827. doi: 10.2196/60827 (PMC11752404; doi:10.2196/60827)
Supplement: Multimedia Appendix 1 [file derma-v8-e60827-s001.docx]

Table 1. Prompts inputted into ChatGPT 3.5, Google Bard, and Bing AI

|  | Diagnosis, treatment, and prognosis prompts inputted into chatbots | | |
| --- | --- | --- | --- |
|  | Condition | Category | Prompt |
|  | Atopic dermatitis | Diagnosis | My 13 month old son with no allergies or medical issues has red dry patches on his stomach, back, face, inside of his elbows, and back of his knees. This started a few months ago. He has no itching. We have tried moisturizer after every bath. Please help provide a diagnosis. |
|  |  |  | I am a 19-year old female with no allergies. I have a scaly, painful, dry, itchy rash that spread from my mouth to my neck, my eyes (causing crusting and soreness), and my right nipple. This has been worsening for months and is rapidly spreading. It is difficult to sleep. My doctor has prescribed me antibiotics and eczema creams and steroids that haven’t helped. I have tried to eliminate allergens. Please provide a diagnosis. |
|  |  | Treatment | Please provide a treatment plan for eczema on my hands which has been present for 4 years. It is itchy and has spread. I’m currently using betamethasone dipropionate. |
|  |  |  | Please provide a treatment plan for eczema on my eyelids and upper lip. I’ve had eczema for 20 plus years that flares on my face twice a year. I’ve tried hydrocortisone cream for 4 months, moisturizer and petrolatum. I use scented soap and I do have stress in my life. I’m 21 weeks pregnant. |
|  |  | Prognosis | How long will it take for my eczema to clear up after treatment with topical steroids? I had a full-body breakout. |
|  | Acne vulgaris | Diagnosis | I am a 20 year old male with recurrent painful deep bump on chin for a couple of months. I have tried puncturing it and using other acne medications, which flattens it for a few weeks but it comes back again for a few months now. Please provide a diagnosis. |
|  |  |  | I am a 22 year old female with bumps around my jawline around the time of my menstrual period. This has been happening for the past year. I tried OTC retinol cream and acne patches, but there has been no improvement. I have no other medical problems. Please provide a diagnosis. |
|  |  | Treatment | Please provide a treatment plan for my acne vulgaris. I have had acne for about 10 years and have tried many different products. My skin is both dry and greasy. |
|  |  |  | I have been treating my acne vulgaris with doxycycline 100 mg every morning and adapalene (0.1%)/benzoyl peroxide (2.5%) every night. My acne cleared at first, but now is no longer responding to this treatment. For the past month, I have been getting acne on my nose and forehead. Can I increase the amount of doxycycline to better control my acne vulgaris? Or, should I switch antibiotics? |
|  |  | Prognosis | I am a young teenager and have been picking at my acne. If I stop picking now, will I still have scars in the future? |
|  | Actinic keratosis | Diagnosis | I am a 55 year old male and have been experiencing scaly spots on my scalp for the last year. I try to pick them off but they just come back. I tried using Head and Shoulders shampoo but have had no improvement. I have no other medical conditions. Please provide a diagnosis. |
|  |  |  | I am a 35 y/o female with a flaky spot on my forehead for the last 6 months. The skin there is a bit thick. I grew up in California with a decent amount of sun exposure and my mother has a history of skin cancer. I have tried moisturizer and OTC hydrocortisone, but it has not improved. I have no other medical conditions. Please provide a diagnosis. |
|  |  | Treatment | I was diagnosed with actinic keratosis on my forehead 7 years ago. 2 years ago, I had more growths frozen off of my face and scalp. My scalp has been crusty. Will photodynamic therapy make my AK worse and will I have to undergo treatment on a regular basis? |
|  |  |  | I was recently diagnosed with actinic keratosis. At my last appointment, I had 14 spots frozen. Are there any other treatment options? |
|  |  | Prognosis | I had my actinic keratosis lesion removed by shave biopsy. I was told it was removed completely, but it is starting to flake again about a week after healing. Is my AK still present? |
|  | Cyst | Diagnosis | I am a 50+ year old male with a bump on my back since age 12. It is soft to the touch. It usually does not bother me, but this last week it became bigger, red, and uncomfortable. I have tried popping it before, but it didn'’t work. Please provide a diagnosis. |
|  |  |  | I am a 35 year old female with a fluid-filled lump on the back of my neck. It is painful and red, with no head on it. It has been present for a couple of weeks. I have Neurocardiogenic syncope, Myalgic Encephalomyelitis, and IBS-C. My current medications are Propanalol and methylphenidate hydrochloride. Please provide a diagnosis. |
|  |  | Treatment | I have has a sebaceous cyst for about 2 months. My physician prescribed me cephalexin 500 mg 4x daily for 7 days. It is currently day 4 and the cyst is much larger, harder, and very red and painful. Does cephalexin work for a sebaceous cyst and how does it work? |
|  |  |  | I had a cyst above my sternum for a couple of months that had not been evolving. I squeezed it last night and keratin came out, but no blood. I am worried it will become infected now. What should I do? |
|  |  | Prognosis | I had an epidermoid cyst that grew inflamed and infected a few weeks ago. I was put on antibiotics. The cyst ruptured about 3 weeks ago with chunks coming out of it, painfully. For the past two weeks now, there is a hole where the cyst was, with pus and blood oozing out every day. Why is it taking so long to heal? Should I go to the ER or urgent care today, see the doctor in 1-‐3 days, follow up in a couple weeks, or treat myself at home? |
|  | Rosacea | Diagnosis | I am a 38 year old female with a past medical history of GERD and anxiety. I am on no medications. I have facial redness on my forehead (between my eyebrows), cheeks, chin, and over my nose. There are some small bumps, it is not itchy, but it feels hot sometimes. It is worse when I am anxious and before my period. Please provide a diagnosis. |
|  |  |  | I am a 40 year old female with idiopathic hypersomnia, ADHD, and major depressive disorder. I am getting flares of facial redness that arise suddenly, feel hot, and make my skin dry and rough. The hotness and redness lasts for a few hours, but my skin is sensitive and rough for several days afterwards. It does not affect my eyes, chin, and forehead. The sun triggers these flares. Some other symptoms I have are hypersomnia, frequent headaches, low energy. Please provide a diagnosis. |
|  |  | Treatment | I was diagnosed with rosacea and my doctor wanted to start me on an oral antibiotic. I am nervous about taking antibiotics. Are there any other topical treatments I can try first? |
|  |  |  | I was diagnosed with ETT rosacea. What are the best treatments? |
|  |  | Prognosis | I was diagnosed with steroid-induced rosacea and prescribed Mirvaso. I continue to experience it redness and burning when I am in warm and humid environments. Is steroid-induced rosacea curable? |
